# Deposition of Crystalline Hydroxyapatite Nanoparticles on Y-TZP Ceramic: A Potential Solution to Enhance Bonding Characteristics of Y-TZP Ceramics

**Published:** 2017-03

**Authors:** Abbas Azari, Sakineh Nikzad Jamnani, Arash Yazdani, Faezeh Atri, Vania Rasaie, Abbas Fazel Anvari Yazdi

**Affiliations:** 1 Associated Professor, Department of Prosthodontics, School of Dentistry, Tehran University of Medical Sciences, Tehran, Iran; 2 Assistant Professor, School of Mechanical Engineering, Shahrood University of Technology, Shahrood, Iran; 3 Assistant Professor, Department of Dental Technology, School of Dentistry, Tehran University of Medical Sciences, Tehran, Iran; 4 Assistant Professor, Department of Prosthodontics, Dental School, Ilam University of Medical Sciences, Ilam, Iran; 5 Department of Biomedical Engineering, Materials and Biomaterials Research Center, Tehran, Iran

**Keywords:** Zirconium Oxide, Hydroxyapatites, Dental Bonding, Microscopy, Electron, Scanning, X-Ray Diffraction, Spectrometry, X-Ray Emission

## Abstract

**Objectives:**

Many advantages have been attributed to dental zirconia ceramics in terms of mechanical and physical properties; however, the bonding ability of this material to dental structure and/or veneering ceramics has always been a matter of concern. On the other hand, hydroxyapatite (HA) shows excellent biocompatibility and good bonding ability to tooth structure, with mechanically unstable and brittle characteristics, that make it clinically unacceptable for use in high stress bearing areas. The main purpose of this study was to introduce two simple yet practical methods to deposit the crystalline HA nanoparticles on zirconia ceramics.

**Materials and Methods:**

zirconia blocks were treated with HA via two different deposition methods namely thermal coating and air abrasion. Specimens were analyzed by scanning electron microscopy, energy dispersive spectroscopy (EDS) and X-ray diffraction (XRD).

**Results:**

In both groups, the deposition techniques used were successfully accomplished, while the substrate showed no structural change. However, thermal coating group showed a uniform deposition of crystalline HA but in air abrasion method, there were dispersed thin islands of HA.

**Conclusions:**

Thermal coating method has the potential to significantly alter the surface characteristics of zirconia. The simple yet practical nature of the proposed method may be able to shift the bonding paradigm of dental zirconia ceramics. This latter subject needs to be addressed in future investigations.

## INTRODUCTION

The popularity of zirconia has increased in the past 10 years. It is suitable for use in many dental applications due to having unique mechanical properties, chemical stability and esthetic characteristics. Stabilized tetragonal zirconia polycrystalline ceramics with 3 mol% of yttria (3Y-TZP) have enhanced mechanical properties and are reliable for posterior multi-unit resin-bonded restorations, implant abutments and intracanal posts [1,2].

Despite good mechanical properties, Y-TZP ceramics have poor bonding ability to tooth structure and veneering ceramics due to their inherent non-polar nature [3]. Various studies tried to improve the bonding properties of dental zirconia by incorporating techniques that primarily intended to change the surface characteristic of Y-TZP ceramics [4–7]. Although these techniques have been successful to some extent, there is still no consensus on the best surface treatment.

Hydroxyapatite (HA) with the formula “Ca_10_(PO_4_)_6_(OH)_2_” known as calcium hydroxide phosphate [8] is the main mineral component of bone and teeth [9,10]. Biological HA has a 1.67 Ca/P ratio [11] which is close to the Ca/P ratio of human enamel and bone (Ca/P =1.58) [12]. It is classified as a biocompatible material [9,13] and it has been shown to be a good alternative to directly bond to hard tissues through a Ca–P-rich layer [14,15] leading to its extensive use in orthopedics, dentistry and maxillofacial reconstructions [13,16–20]. Radiopacity, ideal hardness (i.e. similar to that of natural teeth), good wear properties, high surface tension and excellent wettability are some of the characteristics that make it suitable for use in restorative dentistry [9, 19]. In implant dentistry, HA deposition improves bond strength of bony structures to implant body. However, it may negatively affect the process of osseointegration by HA degradation [21]. Hydroxyapatite has some drawbacks such as brittleness in bulk form, poor tensile strength and low impact resistance [19,20]. Therefore, it may be a good idea to combine the mechanical properties of zirconia with optimal bonding ability of HA.

In order to deposit HA on a surface, different techniques have been used. common methods are sol-gel (chemical solution deposition), ion beam assisted deposition, vacuum deposition, ion-beam sputtering (depositing thin films by ion sputtering), electrophoretic deposition [6,20], high-velocity oxy-fuel spray [10,13], atmospheric plasma spraying, pulsed laser deposition, electrostatic spray deposition and blasting process [14]. It is worthy of note that each method has its own advantages and disadvantages and review of literature revealed no privilege for a specified technique, yet most of them are difficult to use.

In this experimental in vitro study, HA was coated on the surface of Y-TZP ceramic by two sophisticated deposition methods namely air abrasion and thermal coating. The main objective was to find a simple/practical way to coat the Y-TZP ceramics with HA.

## MATERIALS AND METHODS

In this short communication, two experimental coating methods were evaluated namely thermal coating process and air particle abrasion. The Y-TZP dental ceramic blocks (Cercon, Dentsply, Amherst, NY, USA) with dimensions of 4×4×4mm were ground finished by 600-grit silicon carbide abrasive papers (Struers RotoPol 11; Struers A/S, Rodovre, Denmark), cleaned for 10 minutes in an ultrasonic bath (Quantrex 90 WT; L&R Manufacturing Inc., Kearny, NJ, USA) containing ethyl acetate and then air-dried for 30 seconds. For thermal coating method, a slurry of 10g nanoparticulate (less than 100nm) HA powder (Merck, Darmstadt, Germany), 1g of poly vinyl alcohol (Merck, Darmstadt, Germany) and 50cc of distilled water was prepared. The mixture was heated in a magnetic stirrer with hot plate at 100°C with 1000 rpm speed for 30 minutes to form a uniform suspension. The blocks were then soaked in this slurry for 5 seconds at 45° angle and a sintering procedure started according to the following protocol: Room temperature to 300°C (10 minutes), 300°C to 600°C (10 minutes), 600°C to 900°C (30 minutes), 900 to 1200°C (120 minutes) and cooling (annealing) accomplished in a furnace (CWF Furnace; Keison Products, Chelmsford, UK). In air abrasion method, the outer surface of zirconia blocks was blasted with HA nano particles at 4 bar pressure, from a distance of 10mm for 15 seconds using a commercial sandblasting machine (Mestra, Talleres Mestraitua S.L, Sondika Bilbao, Spain) [22]. Then, the procedure was followed by sintering process as described earlier. Scanning electron microscopy was performed (VEGA-XMU; Tescan, Brno, Czech Republic) to investigate (a) microstructural features and (b) elemental distribution in zirconia blocks and to assess the structure and morphology of HA and pattern of coating.

Images were collected in the secondary electron imaging mode with 15kV working voltage and 10–12mm working distance. The composition analysis was performed with an energy dispersive spectroscopy (EDS; Kaker, Ravne na Koroskem, Slovenia) X-ray setup. The EDS provided qualitative data and assessed the presence of different elements in the surface of prepared samples.

The prepared zirconia blocks were examined with X-ray diffraction (XRD) in order to find out the phase of the HA structure. The XRD spectra were collected using a multifunctional Unisantis^®^ diffractometer (XMD300; Unisantis Europe GmbH, Osnabruck, Germany). The Cu K radiation was used at a wavelength of 1.5406^À^ at a current of 0.8 mA and accelerating voltage of 45 kV. Data were collected by the step counting of 0.02, and dwell time of 15 s/step in the range of 10≤2Ø≤100.

Phase identification of zirconia blocks were performed with X’pert High Score 2.2 software (PANalytical, Almelo, Netherlands) with reference to the database supplied by International Center for Diffraction Data (ICDD).

## RESULTS

**Fig. 1:**
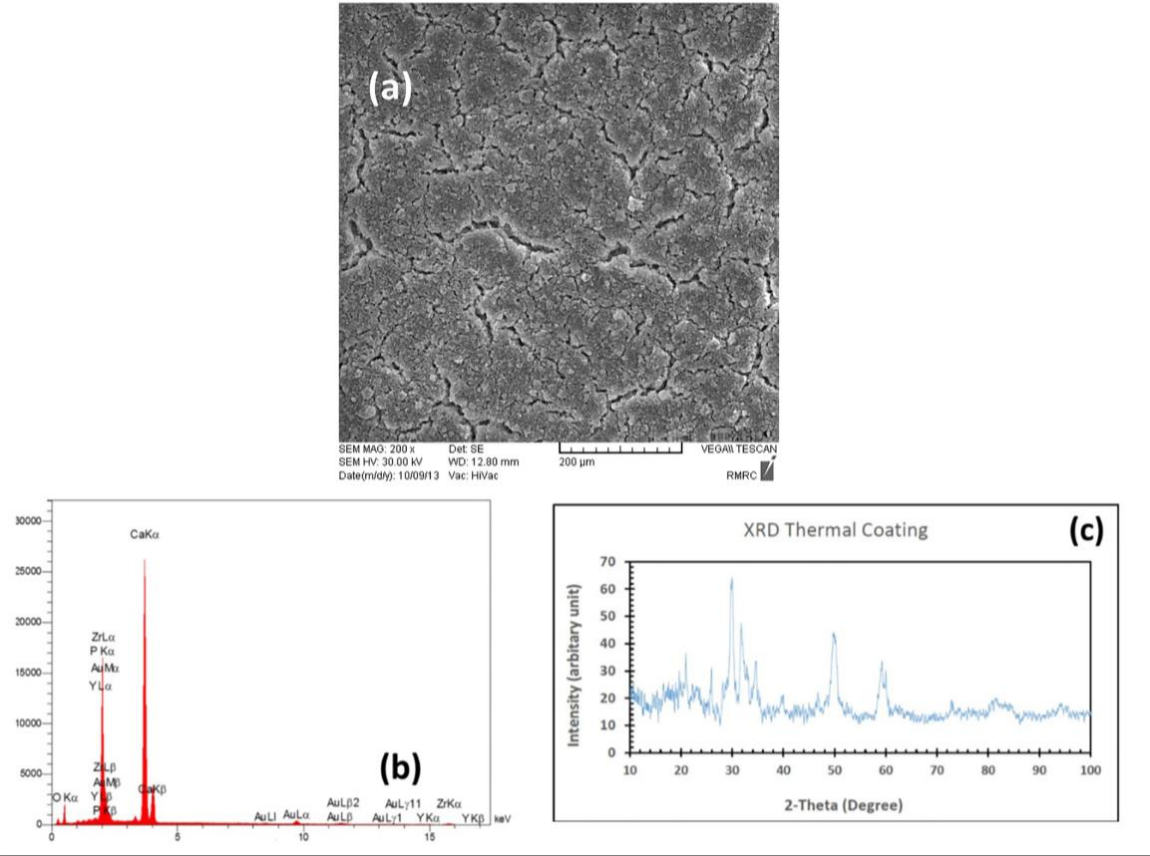
HA coating in TC method: (A) SEM analysis (B) EDS results, (C) XRD pattern

**Fig. 2:**
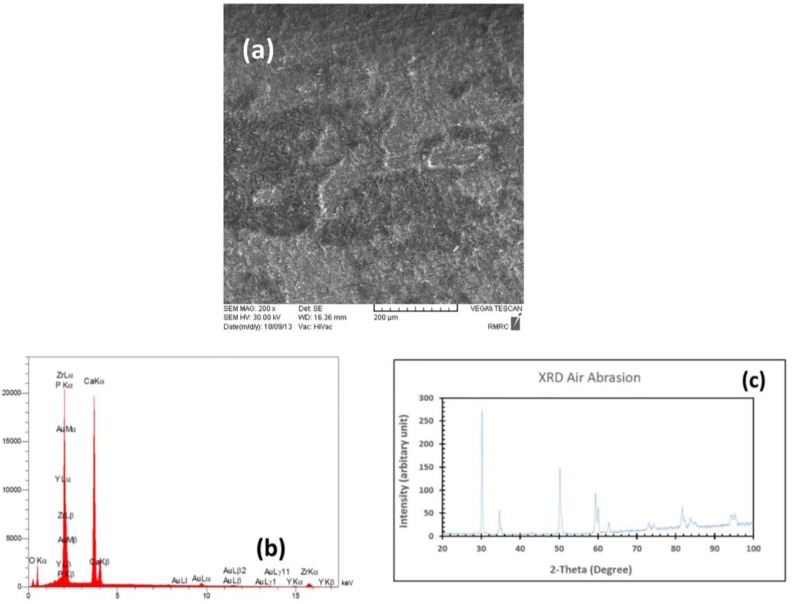
HA coating in AA method: (A) SEM analysis (B) EDS results, (C) XRD pattern

Scanning electron microscopic analysis of the thermally coated block showed a uniform coating on the zirconia surface with various cracking features, which demonstrated a difference in thermal expansion coefficient of zirconia and HA nano powders. In EDS analysis, we saw different elements such as phosphorous and calcium, which demonstrated the presence of HA on the surface of thermally coated sample. The EDS results also showed zirconium and yttrium reflections of substrate. The XRD patterns recorded from thermally coated samples revealed that the crystalline HA formed on the surface of zirconia block. In XRD patterns, there was no reflections belonging to zirconia substrate due to well coating of HA on the surface of zirconia block (Fig. 1). Also, in scanning electron microscopic image related to the air abrasion block, an irregular pattern of dispersed islands of HA coating on the substrate was seen. The EDS results showed reflections of all mentioned elements in thermally coated sample. In XRD pattern of air abrasion sample, we observed that the predominant reflections belonged to zirconia matrix and no reflections were found for HA due to much bigger exposed surface of zirconia substrate (Fig. 2).

## DISCUSSION

In order to exploit the benefit of HA, different coating methods are presented but, there are still no standard guidelines on aspects of specific methods, coating thickness, surface considerations, effects of crystallinity or crystallite size on bond, texture, and porosity. The main purpose of these experimental deposition protocols is to produce HA coatings, which may positively improve the adhesive, compositional, and structural properties of coated substrates.

The temperature used in coating process is very critical. The crystal structure of HA significantly changed at 600–800°C at which semi-crystalline transformation occurred. While in sintering process from 800°C to 1000°C, crystalline cells improved perfectly, from 1000–1200°C, a densification process occurred and the mechanical behavior of HA improved. It was also shown that raising the sintering temperature may increase the stability of HA [23].

Surface area, crystallinity, composition and density of the coating are the factors that have significant influence on the dissolution rate of coating. The crystalline HA coats observed after a post deposition heat treatment of 600°C may significantly reduce the tensile bond strength of the coating [24]. The sol-gel deposition technique is easy but needs further examination to validate its bonding strength to other material surfaces. The bond strength achieved is purely mechanical and and makes it difficult to prevent separation [25,26]. The atmospheric plasma spraying technique is the most widely used method for coating of the implants. This procedure needs high temperature and it has been suggested that the crystalline phase of the HA and/or grain structure of substrate may change during atmospheric plasma spraying [21,27]. The formation of alternative HA phases in this case may result in higher dissolution rates and reduced long term fixation. On the other hand, it was revealed that high thermal deposition processes induced more residual stresses on HA coated surface, which consequently diminished the tensile bond strength.

In the same way, high temperature deposition processes may affect the substrate’s grain structure and adversely reduce its long-term performance. One of the features seen in the high temperature processes like atmospheric plasma spraying is cracking, which may be attributed to the rapid and uncontrolled cooling of the thick HA coatings [28]. The cracks within the HA coating can increase the available surface area that can result in weakening of the HA coating and/or high dissolution rates [21,29]. The use of blasting process in deposition has been discussed in a few studies. In this manner, the HA particles entrained in a compressed air jet are deposited onto substrates, in conjunction with a coincident jet of abrasive particles [21,28–30]. Being a simple procedure, low cost of operational equipment, and low deposition temperature are the main benefits of blast technique. As long as the temperature remains relatively low, HA crystalline phase change would not occur and substrate temperature previously noted for atmospheric plasma spraying will remain in a safe range [30]. Another advantage of lower deposition temperature (<47˚C) in conjunction with lower HA layer thickness is absence of cracks in blast treated HA surface. This feature was confirmed in our study as well. The cracked surface produced during thermal processing of HA in the first experimental block seems to be the result of mismatching of thermal coefficients of HA and zirconia. The thermal expansion coefficients of HA and zirconia are 13.6×10^−6^/K and 10.8 ×10^−6^/K, respectively [31]. However, due to the high thermal stability of zirconia, no changes can be anticipated during heat treatment of HA-coated surface. The XRD test provides definitive structural information and according to our results, both groups revealed HA deposition in the crystalline phase. The XRD indexed on basis of hexagonal crystal system (axis a = b = ∼0.93 nm, axis c = ∼0.68 nm and Y = 120°) of space group of P6_3_/M^20^. The main Bragg peaks in thermal coating group according to JCPDS file No.4-697 [32] observed at 22°, 26°, 31°, 34°, 35°, 40°, 50°and 60°. Due to presence of PVA as binder in apatite lattice, presence of carbonate ions interstitially in its structure, the planar spacing of tetrahedron phosphates increased and broadening of main peaks took place. It has been reported that, at elevated temperature (e.g. 700°C) the carbonate contents began to decompose in gaseous shape like CO_2_ [33,34]. In contrast, in air abrasion group, there was no HA reflection observed in thermal coating group and only main reflections of zirconia substrate could be seen. Both monoclinic (JCPDS No. 65-1024) [35] and cubic (JCPDS No. 49-1642) [36] ZrO_2_ coexist in the XRD pattern at 30°, 35°, 51°, 59°, 60°, 73°, 75°, 82°, 84°, 74° and 75°. Shi et al. [37] confirmed that these reflections belong to Zirconia and show very good crystallinity of substrate.

## CONCLUSION

In the experimental model presented here, two practical methods were assessed namely thermal coating and air abrasion process. In both techniques, the yttria-zirconia substrate was stable. Thermal coating group showed a uniform deposition of crystalline HA but in air abrasion method, there were dispersed thin islands of HA. Thus, thermal coating method had the potential to significantly alter the surface characteristics of zirconia and may be able to shift the bonding paradigm of dental zirconia ceramics. This latter subject needs to be addressed in future investigations.
